# Clinicopathological features of 214 maxillary sinus pathologies. A ten-year single-centre retrospective clinical study

**DOI:** 10.1186/s13005-020-00239-x

**Published:** 2020-10-13

**Authors:** Mario Pérez-Sayáns, José M. Suárez Peñaranda, Juan Antonio Suárez Quintanilla, Cintia M. Chamorro Petronacci, Abel García García, Andrés Blanco Carrión, Pilar Gándara Vila, Yolanda Guerrero Sánchez

**Affiliations:** 1 Health Research Institute of Santiago (IDIS), Oral Medicine, Oral Surgery and Implantology Unit (MedOralRes), Faculty of Medicine and Dentistry, University of Santiago de Compostela, C.P. 15782 Santiago de Compostela, Spain; 2 Pathological Anatomy Service, University Hospital Complex of Santiago (CHUS), C.P. 15782 Santiago de Compostela, Spain; 3 Area of Human Anatomy and Embryology, Faculty of Medicine and Dentistry, University of Santiago de Compostela, C.P. 15782 Santiago de Compostela, Spain; 4 Area oh Human Anatomy and Psychobiology, University of Murcia, Murcia, Spain

**Keywords:** Maxillary sinus pathology, Malignant tumours, Clinic radiological study, Histopathology, Diagnostic indexes

## Abstract

**Background:**

Diagnosis of maxillary sinus pathology must include the clinical radiological study (CRS) and histopathological analysis. The aim of this study is 1) to describe the clinicopathological features of maxillary sinus lesions, obtained successively in a single medical centre over the last 10 years and 2) to determine the sensitivity and specificity for the diagnosis of malignant lesions based exclusively on the CRS.

**Methods:**

It is a single-centre observational retrospective clinical study on patients who attended the University Hospital Complex of Santiago de Compostela (CHUS) with sinus pathologies during the period of 2009–2019.

**Results:**

The sample consisted of 133 men (62.1%) and 81 women (37.9%), with an average age of 46.9 years (SD = 18.8). In terms of frequency, the most frequent pathology was the unspecified sinusitis (44.4%), followed by polyps (18.2%), malignant tumours (9.8%), inverting papilloma (7.5%), fungal sinusitis (4.7%), cysts (3.7%), benign tumours (2.3%), mucocele (2.3%) and other lesions (1.9%). Cysts and benign tumours were diagnosed earliest Vs malignant tumours (65.2 years (SD = 16.1)) were diagnosed the latest (*p* < 0.001). Based only on the CRS for malignancies, diagnostic indexes were 71.4% sensitivity and 97.9% specificity, with a Kappa value of 0.68 with (*p* < 0.001).

**Conclusion:**

Maxillary sinus pathology is very varied with therapeutic and prognostic repercussions. CRS is sometimes insufficient and histopathological confirmation is essential.

## Introduction

The maxillary sinus is a pyramid-shaped structure with its base pointing towards the nasal sidewall and its apex pointing towards the zygomatic process of the maxilla. It contains an ostium which is located towards the cranial side and which connects it to the middle meatus of the nasal cavity, thus enabling the drainage of its content [1]. Its location and distribution facilitates combined sinus pathology (pan-sinus involvement) and conditions the participation of very diverse medical specialists: maxillofacial surgeons, otorhinolaryngologists, odontologists and also allergists [2].

Among the diseases that affect it, anatomical conditions such as aplasia or hypoplasia of the maxillary sinus, non-specific and specific infectious and inflammatory processes, and allergic and tumoral pathologies can be highlighted. Prior to undertaking a surgical intervention on the maxillary sinus and in order to avoid any possible complications, it is very important to evaluate it using diagnostic imaging techniques, by performing a clinical radiological study (CRS) [3] On many occasions, the histopathological study is fundamental in determining a definitive diagnosis, allowing for a correct therapeutic attitude [4].

Inflammatory processes, generically known as sinusitis, are the most frequent sinus pathologies. Sinusitis is classified into five categories: acute maxillary sinusitis, recurring acute sinusitis, subacute sinusitis, chronic sinusitis and acute exacerbation of chronic sinusitis, which may be specific (e.g., fungal) or non-specific. In some cases, patients suffer from chronic asymptomatic inflammatory processes with an unknown aetiology and an abrupt onset which consist of infectious and/or obstructive pathologies. Less frequently cysts, mucoceles, antrochoanal polyps and benign and malignant tumours might appear [2].

The aim of this study is 1) to describe the findings and clinicopathological features of 214 maxillary sinus lesions, obtained successively in a single medical centre over the last 10 years and 2) to determine the sensitivity and specificity for the diagnosis of malignant lesions based exclusively on the CRS.

## Material and methods

### Selections of patients

It is a single-centre observational retrospective clinical study on patients who attended the University Hospital Complex of Santiago de Compostela (CHUS) with sinus pathologies during the period of 2009–2019. All of the procedures performed in this study complied with the ethical standards established by the institutional and research committee and with the Declaration of Helsinki of 1964 and its subsequent amendments. All of the patients gave written or verbal consent to participate in the study and likewise they granted their permission for the research results to be published anonymously. This study received the approval of the Galician Clinical Research Ethics Committee (Ref. 2019/596). It was conducted in accordance with the STROBE guidelines for observational studies.

### Inclusion and exclusion criteria

The inclusion criteria were: patients of any age and gender who attended a medical consultation due to a paranasal sinus pathology and who required at least a confirmatory biopsy or surgical treatment of the lesion. The exclusion criteria were: patients who had not given informed consent, patients with lesions localised in a region other than the maxillary sinus (see ethmoid sinus, sphenoidal or nostril), relapses of previously diagnosed and/or treated lesions, new lesions in the same patient, lesions which affect the maxillary sinus due to loco-regional spreading but which originate in another location, and infectious lesions with odontogenic origin.

### Study variables

The following variables were collected from the study: birth dates, dates of diagnosis, sex, suspected clinical diagnosis, radiological technique (CT or CBCT) and histologically confirmed diagnosis. The clinical diagnosis was determined according the physician’s criteria and was based on clinical and semiological findings and radiological data. The radiological techniques used were cone beam computed tomography (CBCT) and classical computerized tomography on a case-by-case basis. Clinical diagnosis was comprised of: unspecified sinusitis, fungal sinusitis, inverting papilloma, polyp, cyst, mucocele, benign tumours, malignant tumours and others. The histopathological diagnosis allowed for clinical confirmation and made it possible for the origin of the inflammatory/infectious process, the histologic type of the tumour, and/or other clinically undetermined pathologies to be determined in a more specific manner.

### Radiological study

All CBCTs were performed using the same equipment: Planmeca ProMax 3-D Max; Planmeca Oy, Helsinki, Finland). The radiographs were obtained with the patient in the same position and the beam emission parameters were kV = 96, mA = 8, exposure time of 12 s with an image size of 466 voxels (each voxel equals 200 mm). The evaluation software used was Romexis 2.5.1 R (Planmeca Oy, Helsinki, Finland), which allowed observing the data in multiple windows where the axial, coronal and sagittal planes could be visualized in 0.2 mm intervals. The study includes patients from the last 10 years, so the initial radiological tests corresponded to CT that was the device available in the service and once the CBCT was implanted, it was used. All CTs were performed using the Somatom Sensation Open equipment, Siemens, Forchheim, Germany; voxel size: 1.0 × 1.0 × 2.0 mm; scan voltage: 130 kV; and convolution kernel: B30).

### Histopathological study

This was conducted in a routine manner with paraffin inclusion and hematoxylin and eosin stain. Where necessary, multiple sections of each of the blocks were made and PAS and Grocott stains were used in those patients whose clinical suspicion included a micosis. The tumours were classified and studied according the WHO Classification of Head and Neck Tumours and the AJCC criteria (7th and 8th Edition, according the year of diagnosis). When necessary, an immunohistochemical study was performed in order to classify the neoplasms.

### Definitive diagnosis

The definitive diagnosis of tumor pathology based on the CRS is complex and depends on the specialist physician. The decision tree was based on anamnesis, clinical manifestations, and radiological findings. Briefly, the main symptoms of non-tumor pathology included: pain and pressure behind the eyes, nasal discharge and congestion, partial or total loss of the sense of smell, fatigue and general ill feeling, headache or headache, fever, pain throat and drip between the nose and pharynx and sometimes cough. This symptomatology had to be accompanied by the following radiological findings: total or partial veiling of one or more sinuses, intra-sinusal fluid levels, and thickening of the mucosa. In the chronic inflammatory forms, retention cysts, polyps, mucoceles and sometimes images of fungal balls were associated. Tumor pathology was suspected with the existence of: obstructed paranasal sinuses that were not uncovered or with pressure, headache, runny nose and especially bleeding, ulcers inside the nose that did not heal, masses on the face, palate or nose, numbness or tingling in the face, swelling in the eyes or diplopia and pain or mobility in the maxillary teeth. These manifestations had to be accompanied by radiological findings in the form of expansive lesions with soft tissue density, signs of erosion, remodelling and / or destruction of the bone walls. These findings constituted the CRS and conferred a suspected diagnosis that was confirmed by biopsy and histopathological study. Hematoxylin-eosin staining and PAS staining for fungi were routinely performed, and in the case of malignant or highly undifferentiated lesions, immunohistochemistry was used with different epithelial, connective or hematopoietic markers depending on the histological lineage.

The initial suspected diagnosis was only based on the clinical and radiological evaluation (CRS). In case of several possible diagnoses, for the main objective of the study (to differentiate between benign and malignant lesions), it was always considered the worst of suspected diagnoses, evaluating the possibility of malignancies in a dichotomous way (yes or not). The definitive diagnosis was made by the clinical physician based on the results of the anamnesis and the clinical examination, the specific radiological findings on CT/CBCT and the anatomopathological report.

### Statistical analysis

The evaluation has been made basing on the unit of measurement “patient”, since all the patients had only one lesion in the moment of diagnosis. The descriptive statistic was performed using frequencies and percentages for the categorical variables and averages and standard deviations for the quantitative variables. The Kolmogorov-Smirnov test was used to verify the normality of age variance. Contingency tables were drawn up using the Chi-squared test. The analytical statistic was performed by comparing the variables using the ANOVA test for independent samples. Cohen’s Kappa index was calculated to determine the degree of agreement between the clinical and histopathological diagnosis. All of the divergences in which the value of p was less than or equal to 0.05 were considered to be statistically significant. The SPSS 23.0 statistics software was used.

## Results

The analysed sample was comprised of 214 maxillary sinus lesions belonging to 133 men (62.1%) and 81 women (37.9%), with an average age of 46.9 years (SD = 18.8) and an age range from 2.7 to 92.5. A summary of the sample data can be found in Table 1. In terms of frequency, the most frequent pathology was the unspecified sinusitis (44.4%), followed by polyps (18.2%), malignant tumours (9.8%), inverting papilloma (7.5%), fungal sinusitis (4.7%), cysts (3.7%), benign tumours (2.3%), mucocele (2.3%) and other lesions (1.9%). There were no differences regarding gender and clinical diagnoses.

**Table 1:** Summary of the sample data. *Chi-square test for the comparison

**Variable**		**N**	**%**	**Gender distribution**		
**Gender**	men	133	62.1			
	women	81	37.9			
	**Total**	214	100			
**Radiological technique**	CT	24	11.2			
	CBCT	190	88.8			
**Clinical diagnoses**	nonspecific sinusitis	94	43.9			
	polyp	37	17.3			
	benign tumour	23	10.7			
	malignant tumour	19	8.9			
	inverted papilloma	16	7.5			
	fungal sinusitis	10	4.7			
	cyst	12	5.6			
	mucocele	3	1.4			***p*** value*
	**Total**	214	100.0	**Men N (%)**	**Women N (%)**	0.470
**Histopathological diagnoses**	nonspecific sinusitis	95	44.4	55 (57.9)	40 (42.1)	
	polyp	39	18.2	28 (71.8)	11 (28.2)	
	fungal sinusitis	21	9.8	9 (42.9)	12 (57.1)	
	malignant tumour	21	9.8	15 (71.4)	6 (28.6)	
	inverted papilloma	16	7.5	11 (68.3)	5 (31.3)	
	cyst	8	3.7	5 (62.5)	3 (37.5)	
	mucocele	5	2.3	3 (60)	2 (40)	
	benign tumour	5	2.3	4 (80)	1 (20)	
	other	4	1.9	3 (75)	1 (25.0)	
	Total	214	100.0	133 (62.1)	81 (37.9)	

**Fig. 1:**
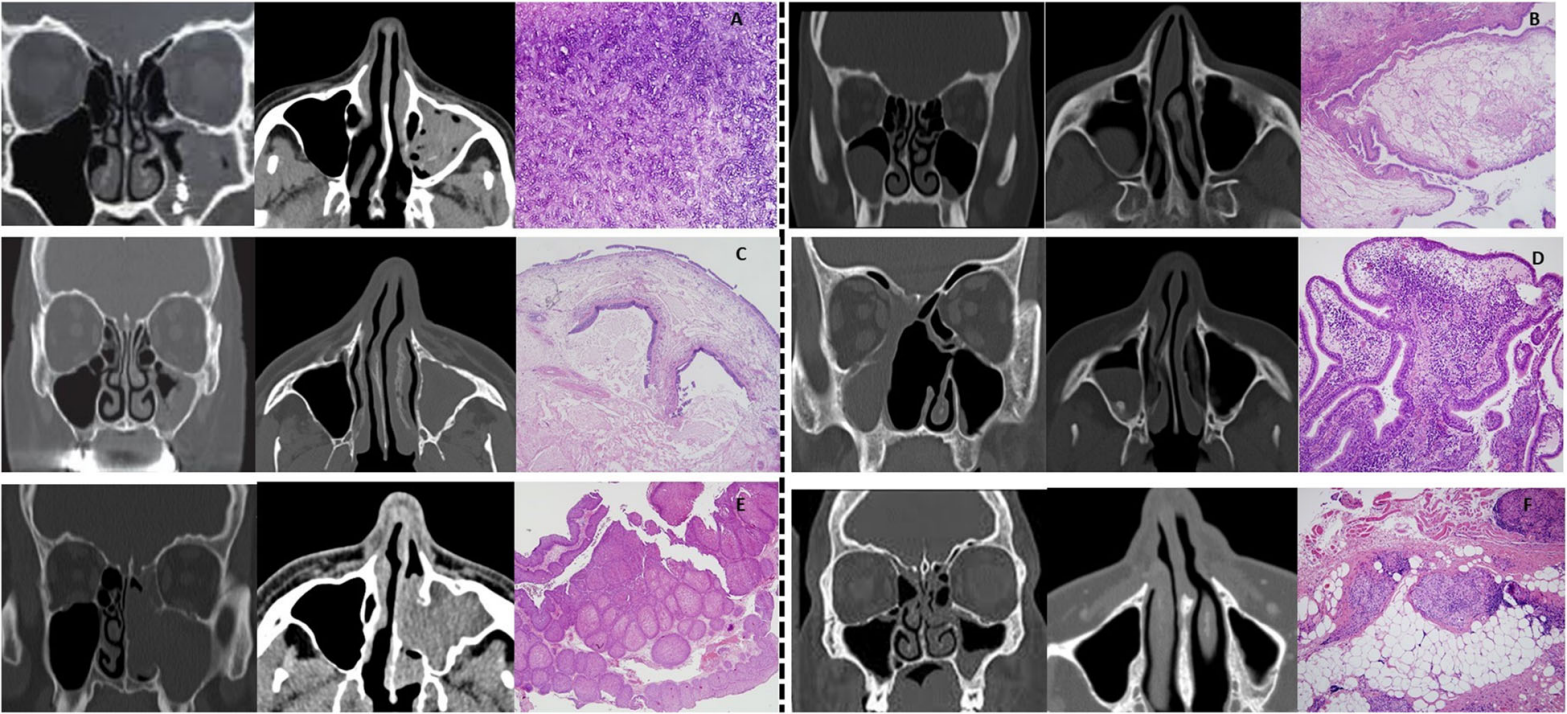
Non-tumoral pathology affecting maxillary sinus, showing the main radiological and the histopathological aspects. **a** Mucormycosis. **b** Retention cyst. **c** Mucocele. **d** Polyp. **e** Inverted papilloma. **f** Non-necrotising granulomatosis. From left to right: coronal/axial sections and histopathology with hematoxylin-eosin

**Fig. 2:**
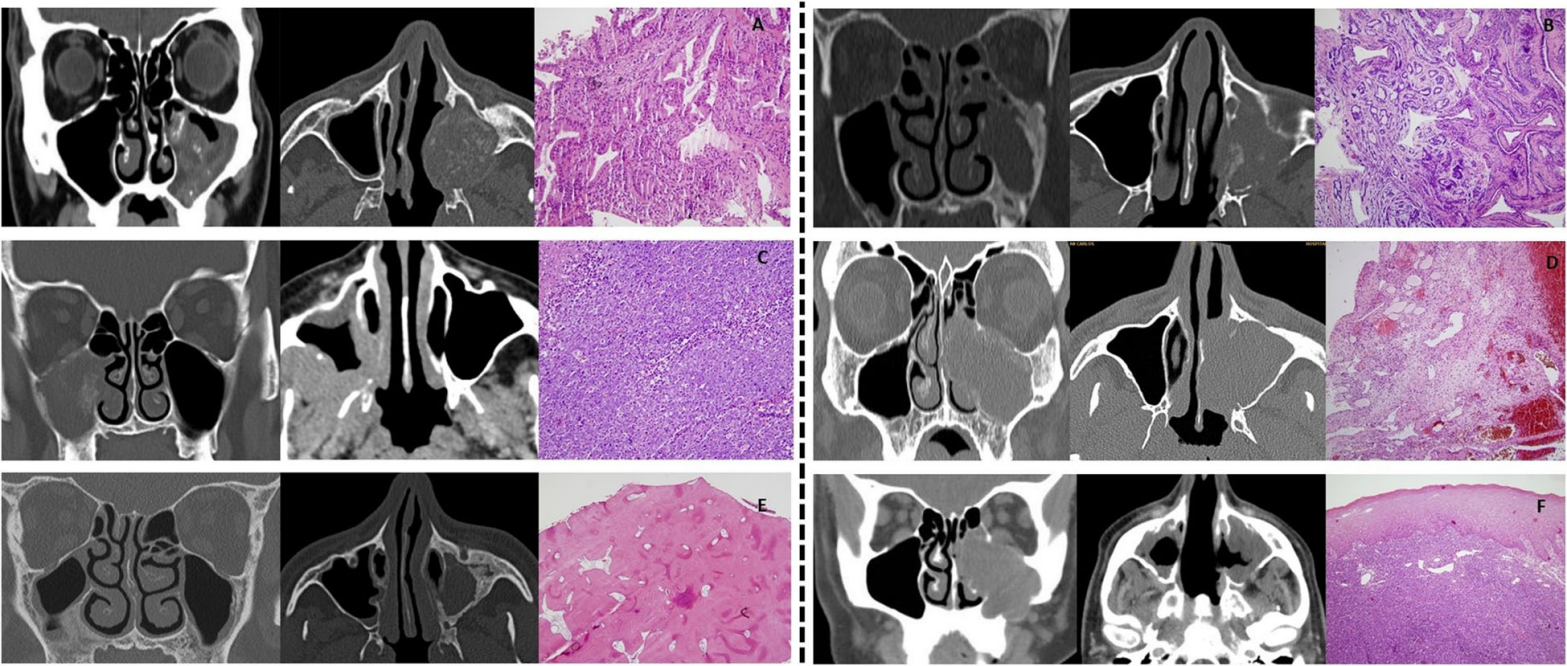
Tumoral pathology (benign and malignant) affecting maxillary sinus, showing the main radiological and the histopathological aspects. **a** Adenocarcinoma. **b** Cystic adenoid carcinoma. **c** Lymphoma. **d** Haemangioma **e** Osteoma. **f** Esthesioneuroblastoma. From left to right: coronal/axial sections and histopathology with hematoxylin-eosin

Fungal sinusitis included: five mucormycosis, five aspergillosis and ten unknown fungus balls (requiring a specific microbiological study). Benign tumours proved infrequent (2.3%) with the following distribution: one fibrolipoma, one cavernous hemangioma, one cystic ameloblastoma and two osteomata. Malignant tumours represented almost 10% of the sample with 52.4% carcinomas, 26.6% adenocarcinomas and 14.3% lymphomas of different histological subtypes and to a lesser extent esthesioneuroblastoma and a metastasis of clear cell renal tumour (Table 2). Other lesions included: a case of maxillary sinus mucosa with fibrosis and vascular congestion (mucosal thickening), a hyperostosis, an organized clot, and a case of non-necrotizing granulomatosis with polyangiitis. Figures 1 and 2, show the most representative lesions of non-tumoral and tumoral pathology affecting maxillary sinus, showing the main radiological and the histopathological aspects.

**Table 2:** Description of histological types of benign and malignant tumours and other lesions

Lesion	Histological Type
**Benign tumours (87.9%)**	1 fibrous lipoma
	1 cavernous haemangioma
	1 cystic ameloblastoma
	2 osteomas
**Malignant tumours (9.8%)**	9 sinonasal epidermoid carcinoma (6 undifferentiated)
	5 enteric-type mucinous adenocarcinomas
	2 diffuse large B-cell lymphoma
	1 NK/T lymphoma, nasal type
	1 cystic adenoid carcinoma
	1 basal-cell carcinoma
	1 carcinosarcoma
	1 Esthesioneuroblastoma
	1 metastasis of clear-cell renal tumour
**Other lesions (2.3%)**	1 Mucosal thickening
	1 Hyperostosis
	1 Organised haematoma
	1 Non-necrotising granulomatosis (granulomatosis with polyangiitis (GPA))

There are statistically significant divergences with regards to the average age of diagnosis of the different diseases (*p* < 0.001). Cysts and benign tumours were diagnosed earliest while the malignant tumours were diagnosed the latest. By applying post hoc tests using the Bonferroni test verified with Sidak, significant differences between some specific diseases were confirmed (Table 3). It should be noted that the average age of patients with malignant tumours was 65.2 (SD = 16.1), higher than the average age of patients with unspecified sinusitis (42.6, SD = 17.7), polyps (42.6, SD = 16.7) and cysts (38.6, SD = 17.2).

**Table 3:** Divergences with regards to the average age of diagnosis of the different diseases. NS: non-significant. N = number. SD = standard deviation

Age	N	Average	SD	Minimum	Maximum	Significant differences
**Non-specific sinusitis**	95	42.6	17.7	2.9	74.9	**Fungal sinusitis** ***p*** **= 0.014** **Malignant tumour** ***p*** **< 0.001**
**Fungal sinusitis**	21	57.5	17.4	20.3	92.5	**Nonspecific sinusitis** ***p*** **= 0.014**
**Inverted papilloma**	16	56.1	11.0	37.8	78.0	NS
**Polyp**	39	42.6	16.7	7.4	71.4	**Malignant tumour** ***p*** **< 0.001**
**Cyst**	8	38.6	17.2	18.8	58.2	**Malignant tumour** ***p*** **= 0.009**
**Mucocele**	5	41.6	26.1	3.6	66.7	NS
**Benign tumour**	5	38.8	24.1	2.7	70.2	NS
**Malignant tumour**	21	65.2	16.1	16.3	87.4	**Nonspecific sinusitis** ***p*** **< 0.001** **Polyp** ***p*** **< 0.001** **Cyst** ***p*** **= 0.009**
**Other**	4	41.2	10.9	32.0	53.7	NS
**Total**	214	47.0	18.8	2.7	92.5	*P* < 0.001

When the degree of agreement and concordance between the clinical and histopathological diagnosis was analysed, a Kappa value of 0.68 with *p* < 0.001 was obtained, reflecting a high, but not infallible rate of clinical success. Among the errors, a case of inverting papilloma and five clinical and radiological benign tumours which ended up being malignant lesions are worth mentioning (Table 4). Based on the clinical and radiology methods alone for the definitive diagnosis of malignancy, we obtained a true positives rate of 78.9%, a false positives rate of 21.1%, a false negatives rate of 8.9% and a true negatives rate of 91.1%. The diagnostic indexes were 71.4% sensitivity and 97.9% specificity. There were no differences regarding the radiological technique (CT or CBCT) and the diagnostic accuracy (*p* > 0.05).

**Table 4:** Degree of agreement and concordance between the clinical and final histopathological diagnosis

		Histopathological Diagnosis									Total
		Nonspecific sinusitis	Fungal sinusitis	Inverted papilloma	Polyp	Cyst	Mucocele	Benign tumour	Malignant tumour	Other	
**Clinical diagnosis**	Non-specific sinusitis	79 (84.0%)	11 (11.7%)	0	4 (4.3%)	0	0	0	0	0	94 (100%)
	Fungal sinusitis	2 (20%)	8 (80%)	0	0	0	0	0	0	0	10 (100%)
	Inverted papilloma	0	0	13 (81.3%)	2 (12.5%)	0	0	0	1 (6.3%)	0	16 (100%)
	Polyp	3 (8.1%)	0	3 (8.1%)	29 (78.4%)	1 (2.7%)	0	0	0	1 (2.7%)	37 (100%)
	Cyst	4 (33.3%)	0	0	0	7 (58.3%)	1 (8.3%)	0	0	0	12 (100%)
	Mucocele	0	0	0	0	0	3 (100.0%)	0	0	0	3 (100%)
	Benign tumour	4 (17.4%)	2 (8.7%)	0	4 (17.4%)	0	1 (4.3%)	5 (21.7%)	5 (21.7%)	2 (8.7%)	23 (100%)
	Malignant tumour	3 (15.8%)	0	0	0	0	0	0	15 (78.9%)	1 (5.3%)	19 (100%)
**Total**		95 (44.4%)	21 (9.8%)	16 (7.5%)	39 18.2%)	8 (3.7%)	5 (2.3%)	5 (2.3%)	21 (9.8%)	4 (1.9%)	214 (100%)

## Discussion

In this study, we analysed the prevalence of different diseases affecting the maxillary sinus for a period of ten consecutive years in a reference university hospital for otolaryngologists and maxillofacial surgeons. The results confirmed the existence of a wide range of diseases with different origins and complexity, not only in terms of diagnosis, but also in terms of prognosis. Clinical and radiological analyses are not always sufficient for an accurate diagnosis. For this reason, the histopathological study is mandatory. A radiological study must be performed using a CT/CBCT and not a panoramic x-ray since it offers less diagnostic sensitivity [3].

Drumond and Cols [5] performed a study exclusively using a CT in order to evaluate the prevalence of different illnesses in 762 maxillary sinuses. They found that 305 examinations (40.02%) were normal and 457 examinations (59.97%) were abnormal: focal mucoperiosteal thickening (21.25%), polypoid lesions (10.76%), chronic sinusitis (7.48%); chronic odontogenic sinusitis (2.29%); neoplasms (2.03%); rhinosinusitis (1.77%); bone lesions, foreign bodies and oroantral fistula in 0.65%; 0.13 and 0.06% respectively. Our study verified that the diagnostic sensitivity using CRS exclusively in maxillary sinus disease is 70%. In terms of using CBCT or CT for diagnostic accuracy, we did not find any difference. This matter has been confirmed by other authors [6].

Rhinosinusitis is defined as the inflammation of the nose and sinuses and it is considered as one of the most frequent diseases in humans, affecting 5–10% depending on the origin of the population. Most of them are inflammatory and/or unspecified allergic and in our paper they represent almost half of all the lesions studied (44.4%). In our study we excluded sinusitis of dental origin, but it must be mentioned that these can account for 10–20% of all chronic sinusitis More than 95% of the sinusitis (including unspecific and fungal) were properly diagnosed using CRS [7].

The second most frequent disease was polyps (18.2%), included within what is known as antrochoanal polyps (ACP). ACPs are benign lesions which emerge in the maxillary sinus mucosa and reach the choana, and the main symptom of these is nasal obstruction [8]. Men tend to suffer more from this disorder (18.2%) and the average age of diagnosis is 42.6 years, however there is a wide age range from 7.4 to 71.4 years. 7.8% of our cases were under the age of 18, coinciding with the results of Segal et al. [9]. 80% were diagnosed using CRS, however 8.1% turned out to be inverted papillomas, so the pathological anatomical confirmation is very important due to the different nature of this lesion and its tendency to relapse if not properly removed [10]. No differences have been found in term of gender.

A specific kind of sinusitis is the fungal sinusitis, which represented 9.8% of cases in our study. In the CRS these are described as fungus balls in order to differentiate them from mycetomas, as a tropical disease [11–15]. Mucormycosis [16] and aspergillosis [17] are among the most frequent specific entities. According to Pagella and Cols [12] only 34.5% of the cases with fungal hyphae present in the biopsy were microbiologically positive in the culture. 100% of the lesions with non-invasive fungus balls presented hyphae in the anatomopathological study. Non-invasive lesions are more serious with an associated risk of systemic involvement [14], however, in our study we did not find any such case. According to Lim and Cols [15] the average age of diagnosis is 63.1 years with significant female predominance; this data was confirmed by Yoon et al. in a study of 538 sinus fungus balls [13]. In our study, 57.1% were women and the average age was 57.5 years. The most common etiologic organism of this disease is Aspergillus fumigatus, an ubiquitous dimorphic fungus that causes several types of lung disease. In non-invasive aspergillosis, the fungus colonizes a pre-existing cavity and forms a fungus ball consisting of compact concentric hyphae. The demonstration of hyphae and the lack of eosinophils in the mucus of these patients differentiates this condition from allergic fungal sinusitis. Its infection can coexist with other processes, such as non-specific bacterial sinusitis and less frequently with actinomyces [18]. Mucormycosis, on the other hand, is an angioinvasive fungal infection associated with high morbidity and mortality. A change in the epidemiology of mucormycosis has been observed over the past few years with an increased incidence worldwide [16].. According to Celis et al. [19] the average age was 53.14 years. Four patients were immunosuppressed and three were immunocompetent. Indolent mucormycosis is a new and emerging clinical entity in immunosuppressed and also immunocompetent patients. The single paranasal presentation is infrequent and should not be overlooked as a differential diagnosis.

Sinonasal tumours are rare neoplasms. The diagnosis and treatment of these tumours poses several challenges due to their low incidence, histological diversity, and the production of nonspecific symptoms in the early stages. In this study, the neoplastic pathology of the maxillary sinus, contrary to expectations, was very frequent, with the malignant tumours (9.8%) surpassing the benign ones (2.3%). The most common types were epidermoid carcinoma and adenocarcinoma (Table 3). More than 80% of the carcinomas were poorly differentiated, coinciding with the data from Banuchi and Cols’ [20] and Santos and Cols’ [21] studies. The CRS does not allow for the diagnosis or pattern of invasion to be clearly determined [22]. The therapeutic management is complex and requires surgery, chemotherapy and radiotherapy, offering a reserved prognosis [23] Other histological varieties were lymphomas, mostly histological subtype B, although we also found a case of nasal type T/NK lymphoma, the most common varieties in this location. Within the head and neck, the majority of the extranodal lymphomas emerge in the Waldeyer ring; comprised of the adenoid tonsils, Eustachian tonsils, palatal tonsils, and lingual tonsils, although other commonly involved sites include the nasal cavity and sinuses [24, 25]. An important histological subtype is the adenoid cystic carcinoma (Table 3), (old cylindroma) which, although it tends to have a predilection for the head and neck, actually represents less than 2% of malignant tumours of the head and neck and 5 to 15% of paranasal sinus although it has a high capacity for local infiltration [26, 27]. Esthesioneuroblastoma is a rare neoplasm that arises from the nasal cavity’s olfactory neuroepithelium. It shows bimodal age distribution, with peaks from the second to the third, as well as from the sixth to the seventh, decades of life [28]. In our case it was a 39-year-old male. Metastases are rare, but they can occur. The most common metastases originate in the kidney, breast, thyroid and prostate, although they have been described as originating in multiple locations [4]. In our series, we found a metastasis of clear cell renal carcinoma, which appears to be the histological subtype with greatest predilection to cause metastases in the maxillary sinus [29, 30].

The inverted papilloma is a rare neoplasm preferably located in the lateral nasal wall, it is characterized by its relapse tendency, and its potential transformation into malignant neoplasms [31]. In our study, it was the fifth most frequent pathology present in 7.5% of the cases and it was more prevalent in men (68.3%) with an average age of 56 years. Although lesion recurrences were excluded from the study, we observed a recurrence in more than 60% of the cases. Diaz Molina et al. [31], found similar results in which 62% of cases were male with an average age of 58 years. Adriansen and Cols [32]., uncovered similar results with an average age of 47.8 years and 71.4% of cases were male. The CRS follow-up of these patients should be very close.

A more varied group of diseases of high diagnostic complexity by CRS are those known as cysts, pseudocysts and mucoceles. Basically, mucoceles are (cyst-like) structures covered with epithelium and filled with mucin, produced by the obstruction of the drainage ostium, due to their destructive capacity these usually cause pain [33] Retention cysts, on the other hand, have a linear epithelial cystic structure that does not cause destruction and these are caused by alterations in the homeostatic balance of the mucosa. The average patient age is around 40 years old in both cases and predominantly male, coinciding with Veltrini and Cols’ findings [34].

Benign tumours were the least prevalent group: fibrolipoma, cavernous haemangioma, cystic ameloblastoma, and two osteomata. Benign tumours presented greater diagnostic difficulty using CRS, 17.4% were non-specific sinusitis, 17.4% polyps, 8.7% fungal sinusitis, 4.3% mucocele and 8.7% other lesions, but the most remarkable fact is that almost 22% of clinically suspected lesions were confirmed as malignant neoplasms (Table 3). Lipomas in the maxillofacial territory are rare, and the site of predilection is the mucosa of the oral cavity as we have previously reported [35], nonetheless, the maxillary sinus can host these as well [36]. Here we present a case of a seven-year-old boy, histological subtype fibrolipoma. Haemangioma is a common benign vascular lesion of the head and neck region. The mucous membranes of the nasal cavity and paranasal sinuses are rarely implicated [37], although the cavernous variety has been previously published [38]. In our series there is only one case, a 38-year-old male, whose CRS did not provide accurate information. Odontogenic tumours are lesions that are derived from the epithelium, ectomesenquim or mesenchymal components that form part of the dental development apparatus. These tumours are located centrally (intraosseous) within the jaws or peripheral (extraosseous), located inside the gingival mucosa, alveolar or less frequently the maxillary sinus [39]. The ameloblastoma is a common odontogenic tumour of the jaw which is comprised of three variants: conventional (solid), unicystic and peripheral ameloblastomas. The unicystic ameloblastoma in the maxillary sinus is very rare; we present a case of a 73-year-old male, which differs from the few cases published in the literature in which the age of diagnosis is younger [40]. Osteoma is a benign tumour composed of well-differentiated bone tissue with laminar structure, located in bones or soft tissues. Osteomata mainly occur in the head and neck region, especially in the jaw and paranasal sinuses, which is the most common benign tumour of the paranasal sinuses [41, 42].. Among our cases, there was only one 70-year-old male with typical histopathological features who was diagnosed in the CRS.

We have included a group of lesions, framed as “other lesions”: 1) opacification, fibrosis and mucosal thickening, which is the most frequent radiological finding of the maxillary sinus [41], but which in our case was diagnosed as a polyp in the CRS; 2) a hyperostosis, which was confused with a benign tumour and which tend to be normal anatomical variations [43]; 3) an organized hematoma that was suspected to be malignant in a patient with chronic myeloid leukemia and that has been recently described [44]; 4) non-necrotizing granulomatous disease in a 33-year-old woman as part of a clinical picture of eosinophilic granulomatosis with polyangitis, an autoimmune systemic disease manifesting as asthma, recurrent sinusitis and peripheral eosinophilia [45].

## Conclusions

The pathology of the maxillary sinus is very varied and it has very different therapeutic and prognostic repercussions, so its correct characterization is essential. As we have proven, CRS is sometimes insufficient and histopathological confirmation is essential.

## Data Availability

The datasets used and/or analysed during the current study are available from the corresponding author on reasonable request.
